# Social Network Analysis of COVID-19 Sentiments: Application of Artificial Intelligence

**DOI:** 10.2196/22590

**Published:** 2020-08-18

**Authors:** Man Hung, Evelyn Lauren, Eric S Hon, Wendy C Birmingham, Julie Xu, Sharon Su, Shirley D Hon, Jungweon Park, Peter Dang, Martin S Lipsky

**Affiliations:** 1 College of Dental Medicine Roseman University of Health Sciences South Jordan, UT United States; 2 Department of Orthopaedics University of Utah Salt Lake City, UT United States; 3 George E Wahlen Department of Veterans Affairs Medical Center Salt Lake City, UT United States; 4 Department of Occupational Therapy & Occupational Science Towson University Towson, MD United States; 5 David Eccles School of Business University of Utah Salt Lake City, UT United States; 6 Department of Educational Psychology University of Utah Salt Lake City, UT United States; 7 Division of Public Health University of Utah Salt Lake City, UT United States; 8 Department of Biostatistics Boston University Boston, MA United States; 9 Department of Economics University of Chicago Chicago, IL United States; 10 Department of Psychology Brigham Young University Provo, UT United States; 11 College of Nursing University of Utah Salt Lake City, UT United States; 12 Department of Electrical & Computer Engineering University of Utah Salt Lake City, UT United States; 13 School of Computing University of Utah Salt Lake City, UT United States; 14 International Business Machines Corporation Poughkeepsie, NY United States

**Keywords:** COVID-19, coronavirus, sentiment, social network, Twitter, infodemiology, infodemic, pandemic, crisis, public health, business economy, artificial intelligence

## Abstract

**Background:**

The coronavirus disease (COVID-19) pandemic led to substantial public discussion. Understanding these discussions can help institutions, governments, and individuals navigate the pandemic.

**Objective:**

The aim of this study is to analyze discussions on Twitter related to COVID-19 and to investigate the sentiments toward COVID-19.

**Methods:**

This study applied machine learning methods in the field of artificial intelligence to analyze data collected from Twitter. Using tweets originating exclusively in the United States and written in English during the 1-month period from March 20 to April 19, 2020, the study examined COVID-19–related discussions. Social network and sentiment analyses were also conducted to determine the social network of dominant topics and whether the tweets expressed positive, neutral, or negative sentiments. Geographic analysis of the tweets was also conducted.

**Results:**

There were a total of 14,180,603 likes, 863,411 replies, 3,087,812 retweets, and 641,381 mentions in tweets during the study timeframe. Out of 902,138 tweets analyzed, sentiment analysis classified 434,254 (48.2%) tweets as having a positive sentiment, 187,042 (20.7%) as neutral, and 280,842 (31.1%) as negative. The study identified 5 dominant themes among COVID-19–related tweets: health care environment, emotional support, business economy, social change, and psychological stress. Alaska, Wyoming, New Mexico, Pennsylvania, and Florida were the states expressing the most negative sentiment while Vermont, North Dakota, Utah, Colorado, Tennessee, and North Carolina conveyed the most positive sentiment.

**Conclusions:**

This study identified 5 prevalent themes of COVID-19 discussion with sentiments ranging from positive to negative. These themes and sentiments can clarify the public’s response to COVID-19 and help officials navigate the pandemic.

## Introduction

The outbreak of coronavirus disease (COVID-19) upended people’s lives worldwide. COVID-19 is caused by severe acute respiratory syndrome coronavirus 2 (SARS-CoV-2), a novel human pathogen that virologists believe emerged from bats and eventually jumped to humans via an intermediary host [1]. Clinical manifestations range from mild or no symptoms to more severe illness that may result in pulmonary failure and even death [2].

On March 11, 2020, the World Health Organization (WHO) declared COVID-19 a pandemic [3]. By June 23, the WHO reported 8,993,659 confirmed COVID-19 cases globally, and 469,587 deaths [4], and the Centers for Disease Control and Prevention (CDC) reported more than 2 million confirmed cases in the United States and more than 120,000 deaths [5]. These numbers illustrate how swiftly an emerging infection can spread.

For a novel virus without an available vaccine or highly effective antiviral drug therapy, community mitigation represents one strategy to slow the rate of infections. Community mitigation for COVID-19 consists of physical distancing including closing schools, bars, restaurants, movie theatres, and encouraging businesses to have their employees work from home. Large public gatherings such as festivals, graduations, and sporting events are discouraged or banned. The economic impact of mitigation devastated numerous businesses, while in the United States alone, over 40 million people filed initial unemployment claims [6].

Mitigation can also incorporate stay-at-home orders except for managing essential needs and for workers with an essential job. The isolation associated with mitigation is linked to stress, depression, fear and denial, exacerbation, and posttraumatic stress disorder (PTSD) [7-9]. Extended social isolation can exacerbate existing mental health problems, anxiety, and angry feelings. People in isolation may have also lost social support from families and friends. Resentment and resistance to these changes in daily life is becoming increasingly evident [10].

To inform personal decisions about health issues, individuals often use the media as a source of up-to-date information [11]. The intensity of information may make this particularly true for the COVID-19 pandemic. Despite daily information, major questions remain about viral spread, postrecovery immunity and drug therapy [12]. To interpret what may seem as information overload, many individuals turn to social media for clarification. There they can find an abundance of pandemic-related discussion about the economy, school closure, lack of medical supplies and personnel, and social distancing.

Unfortunately, media messaging may not always align with science and misinformation, baseless claims, and rumors can spread quickly. For example, commentary that SARS-CoV-2 originated as a Chinese conspiracy increased xenophobic sentiment toward Asian Americans [13]. The impact and speed of the COVID-19 pandemic mean that understanding public perception and how it affects behavior is critically important. Failing to do so creates both time and opportunity costs.

In contrast to traditional news reporting, which often takes weeks, social media messages are available in virtually real time [14]. These sources offer an opportunity for earlier insights into the public’s reaction to the pandemic. Among social media sites, Twitter is the most popular form of social media used for health care information [15]. Previous studies indicate that Twitter can yield important public health information including tracking infectious disease outbreaks, natural disasters, drug use, and more [16].

Despite the importance of understanding the public reaction to COVID-19, gaps in the understanding of COVID-19–related themes remain. To address this gap, this study conducted a social network analysis of Twitter to examine social media discussions related to COVID-19 and to investigate social sentiments toward COVID-19–related themes. Study goals were twofold: to provide clarity about online COVID-19–related discussion themes and to examine sentiments associated with COVID-19. Findings from this study can shed light on unnoticed sentiments and trends related to the COVID-19 pandemic. The results should help guide federal and state agencies, business entities, schools, health care facilities, and individuals as they navigate the pandemic.

## Methods

### Data Source

Twitter is a microblogging and social network platform where users post and interact with messages called “tweets.” With 166 million daily users [17], Twitter is a valuable data source for social media discussion related to national and global events. This study collected data from the Twitter website by applying machine learning (ML) methods used in the field of artificial intelligence. To be representative of the population, this study examined tweets originating from the United States during the 1-month period from March 20 to April 19, 2020. The study excluded tweets written in languages other than English or with geolocation outside of the United States. A modified Delphi method was used to identify potential keywords for the Twitter search. Specifically, one author reviewed the literature to identify potential key words. These keywords were then circulated among the other authors for feedback and to solicit additional terms. After two cycles, consensus was obtained for the 13 keywords (Table 1) used to search Twitter posts related to COVID-19. Data extracted from Twitter consisted of the following: date of post, username, tweet content, likes count, replies count, retweets count, place, and mentions. The collected tweet set did not include the content of retweets and quoted tweets.

**Table 1:** Keywords for Twitter post search (N=1,001,380).

Keyword	Frequency, n
Coronavirus	250,849
Covid	340,522
COVID-19	108,035
SARS-CoV-2	670
Stay home	47,772
covid19	134,773
lockdown	46,452
shelter in place	9967
coronavirus truth	1694
outbreak	16,045
pandemic	135,879
quarantine	325,770
social distancing	65,725
hoax	14,703
be kind	4071
health heroes	88
ppe	48,710
isolation	22,459
homeschooling	3271
school cancelled	50
online teaching	475

### Data Analyses

This study applied natural language processing, a form of ML, to process the tweets. To increase precision and to facilitate content analysis of the tweets, background noise such as URLs, hashtags, stop words, and tweets with less than three characters were removed. Lemmatization, a process of reducing the inflectional forms of words to a common root or a single term [18], was applied to the tweets as part of data cleaning. Topic modeling using Latent Dirichlet Allocation (LDA) [19] was used to extract the hidden semantic structures in the tweet posts. The LDA is an unsupervised ML method suitable for performing topic modeling. It groups common words into multiple topics and works well with short or long texts. The study employed several sets of topic modeling, with each set containing 5 to 10 topics, with the authors selecting the topic sets that looked more sensible and interpretable. Following the selection of a set of topics, the authors reviewed the top 10 words from each topic and by consensus developed a theme for each of the topics. Sentiment analysis using Valence Aware Dictionary and sEntiment Reasoner (VADER) determined whether the tweet posts expressed positive, neutral, or negative sentiments, as well as the degree of sentiments (also known as compound score or sentiment score). Sentiment scores were calculated for each theme, ranging from –1 to 1, with –1 representing the most negative sentiment and 1 representing the most positive sentiment. VADER, a sentiment analysis tool based on lexicons of sentiment-related words, allows automatic classification of each word in the lexicon as positive, neutral, or negative. Positive sentiment was categorized by having sentiment scores ≥0.05; neutral sentiment was categorized by sentiment scores between –0.05 and 0.05; and negative sentiment was defined by having sentiment scores ≤–0.05. A random sample of 300 tweets were manually coded as having positive, neutral, or negative sentiments by the investigators, and checked against the machine’s output of sentiment classifications. Sensitivity and specificity were then calculated to evaluate the quality of the work done by the machine. The distribution of the sentiment and user social network connectivity were examined across themes. Centrality measures assessed importance, influence, and significance of the social network themes. Further analyses examined average sentiments across different states in the United States. Python (Version 3.8.2) [20] and R (Version 3.6.2; R Foundation for Statistical Computing) were used to collect and process the data as well as to conduct the data analyses.

To enable research reproducibility and ensure completely transparent methodologies and analyses, all of the computer code for data collection, data analyses, and figure generation are provided in Multimedia Appendix 1. Readers interested in replicating this study or conducting a similar study can reference these computer codes. Due to the large amount of data that needs to be processed and analyzed, using a supercomputer or other high-performance computing resources is recommended.

## Results

**Figure 1:**
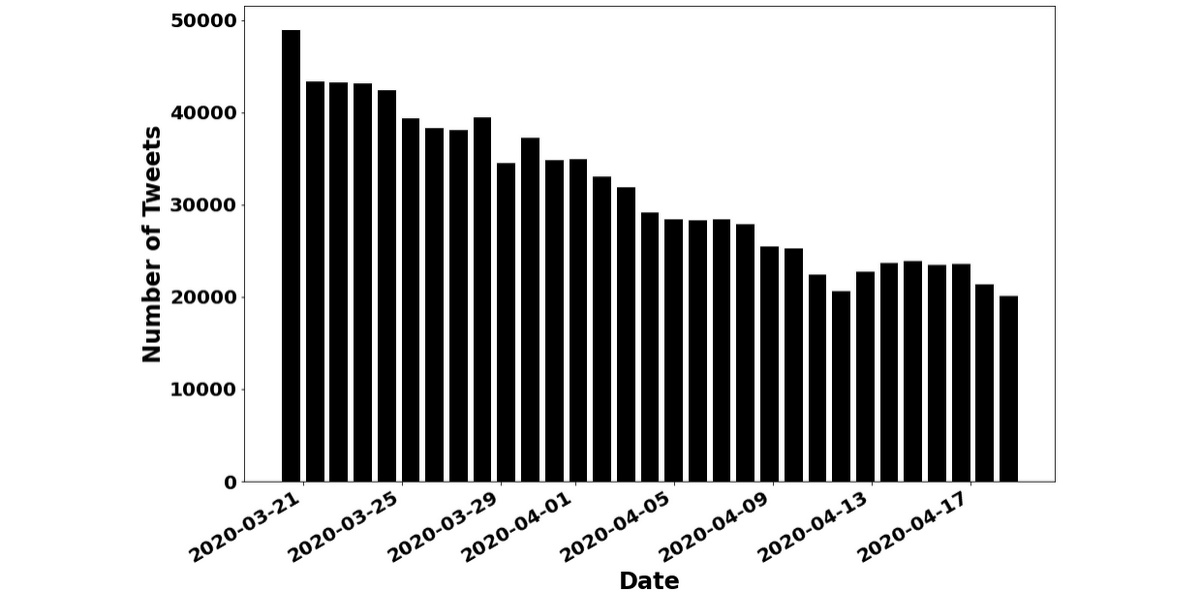
Number of tweets related to coronavirus disease (COVID-19) from March 20, 2020, to April 19, 2020.

**Figure 2:**
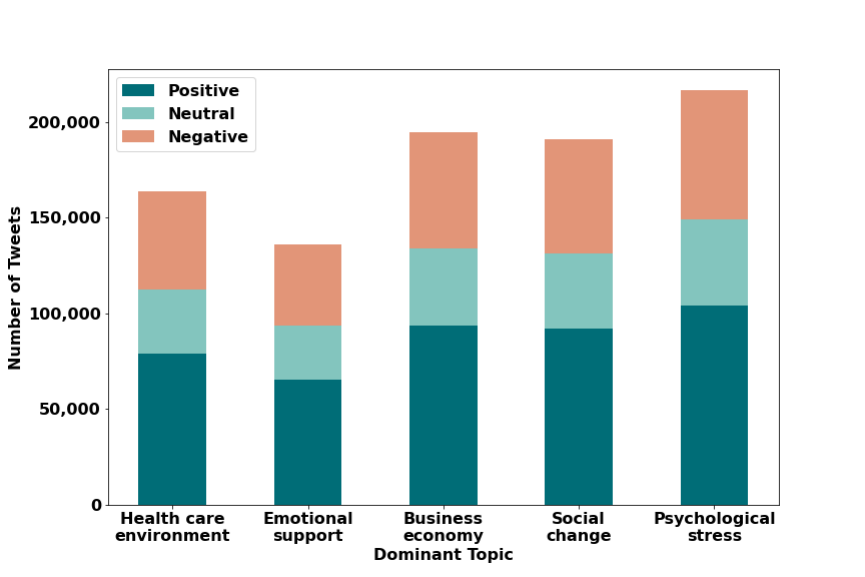
Frequency distribution of dominant topic tweets across sentiment types.

**Figure 3:**
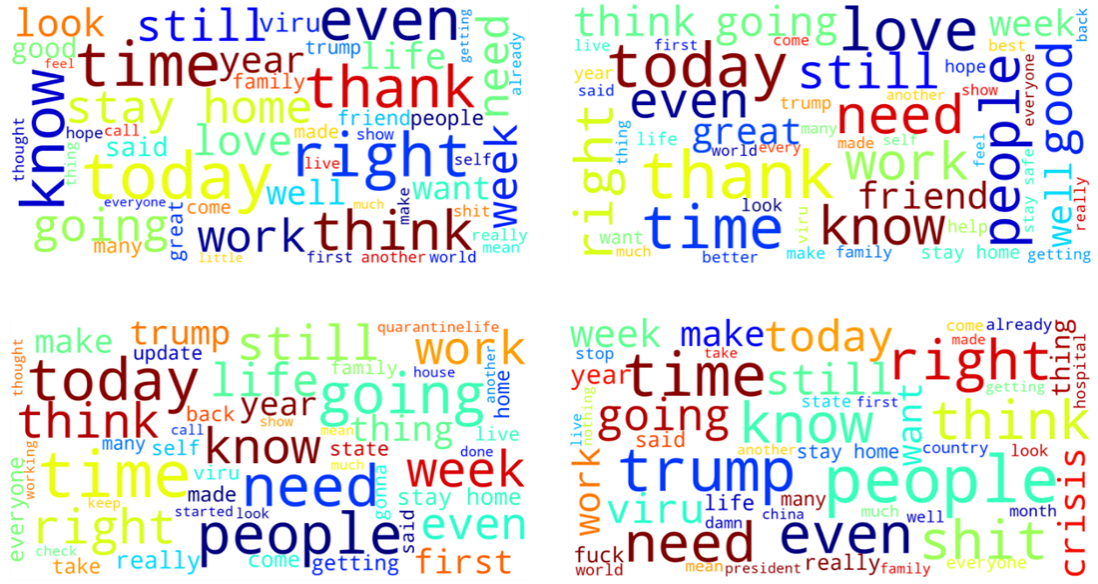
Word clouds showing the most frequently used word stems across Twitter users' post descriptions related to coronavirus disease (COVID-19). The upper left image is a word cloud formed from all tweets, while the upper right image is formed from tweets of positive sentiment. The lower left image is formed from tweets of neutral sentiment, while the lower right image is formed from tweets of negative sentiment.

During the 1-month data collection period, a total of 1,001,380 tweets were retrieved from 334,438 unique Twitter users, representing 12,203 cities within the 50 states in the United States and the District of Columbia. Figure 1 displays the number of tweets related to COVID-19 from March 20 to April 19, 2020. There was a gradual decline in the number of tweets over time. There was a total of 14,180,603 likes, 863,411 replies, 3,087,812 retweets, and 641,381 mentions. After accounting for background noise and performing lemmatization, there was a total of 902,138 tweets remaining, in which sentiment analysis classified 434,254 (48.2%) tweets as having positive COVID sentiment, 187,042 (20.7%) as having neutral COVID sentiment, and 280,842 (31.1%) as having negative COVID sentiment (Figure 2). Overall, positive tweets outweighed negative tweets with a ratio of 1.55 to 1. The most positive sentiment words consisted of “today,” “love,” “work,” “great,” “time,” “thank,” “think,” “right,” and “know,” whereas negative sentiment words related to “people,” “Trump,” “think,” “right,” “time,” “need,” “virus,” and “shit” (Figure 3 and Table 2). Examples of tweets expressing positive, neutral, and negative sentiments are displayed in Table 3. Sensitivity of the positive sentiments was 89.3% and specificity was 77.3%.

**Table 2:** The most frequently used word stems across Twitter users’ post descriptions related to coronavirus disease (COVID-19) by sentiment.

All tweets	Positive sentiment	Negative sentiment	Neutral sentiment
today	thank	people	time
time	today	time	today
right	time	trump	need
even	love	even	people
know	people	know	going
thank	need	right	week
think	know	need	know
stay home	right	think	right
going	work	shit	still
work	even	going	think
still	still	today	work
week	good	still	life
need	going	virus	even
love	think	week	thing
look	great	work	trump
year	friend	crisis	first
life	week	make	year
well	well	want	make
want	stay home	year	really
good	want	thing	stay home
said	life	really	everyone
virus	hope	said	come
people	look	fuck	getting
many	trump	life	made
friend	help	stay home	take
great	year	many	self
family	make	everyone	home
trump	family	state	back
come	better	stop	state
made	best	country	update
really	stay safe	come	virus
make	virus	world	family
mean	many	hospital	live
show	said	much	said
shit	made	look	gonna
self	getting	damn	many
hope	thing	mean	quarantinelife
thought	live	already	started
world	everyone	month	mean
first	world	well	call
another	really	president	much
live	show	china	show
call	come	family	house
thing	self	take	look
already	feel	made	thought
everyone	first	getting	working
much	much	live	check
getting	back	first	done
feel	every	another	keep
little	another	nothing	another

**Table 3:** Examples of tweets expressing positive, neutral, and negative sentiments about coronavirus disease (COVID-19).

Positive sentiments	Neutral sentiments	Negative sentiments
*great zoom call this morning. helping a handfull of clients and business associates. seems like a monday. quarantine won't stop business from happening. it just might look a little different.* *this quarantine is doing me good on the writing* *finding food pleasures in a time of #covid19 crisis* *on the bright side I’ll come out of this quarantine with gorgeous and glowing skin* *thank you to all of the amazing nurses who are putting themselves on the frontline every day, being of great service to those who are in need during this pandemic. thank you for all you do* *woke up to excellent news! i knew a person who was battling covid-19, on a ventilator in a different state. she is my age. she was discharged from the hospital yesterday and is now home! hooray!*	*this is why social distancing is so important* *day 9 of quarantine: i bought legos.... they should be here by friday* *what i have learned from quarantine is that people like to do push ups and take shots* *what if someone has #coronavirus with no symptom, can they donate blood?* *i keep a list of all the people i have come in contact and the places i've been to since the social distancing and shelter at home started* *praying my friend recovers from covid19*	*so sick of covid-19, corona virus !!! tired of social distancing. tired of it all.* *the only thing covid 19 will accomplish is turning a bunch of medical professionals into functioning alcoholics* *had my first quarantine related super hardcore anxiety attack today* *the saddest moment during the covid-19 pandemic was when muni stopped running the 38r* *i should add that dad died before tests were available, and while his doctor said he believed it was covid-19, he could not definitely say that it was so. but this, too, is a failure of governance, given that we knew it was a threat in January* *this is possibly the most insensitive (and couldn’t possibly be true) ads i’ve seen in awhile. taking a victory lap over a financial crisis (because of a viral pandemic which is costing people much worse things than their finances) is gross.*

**Figure 4:**
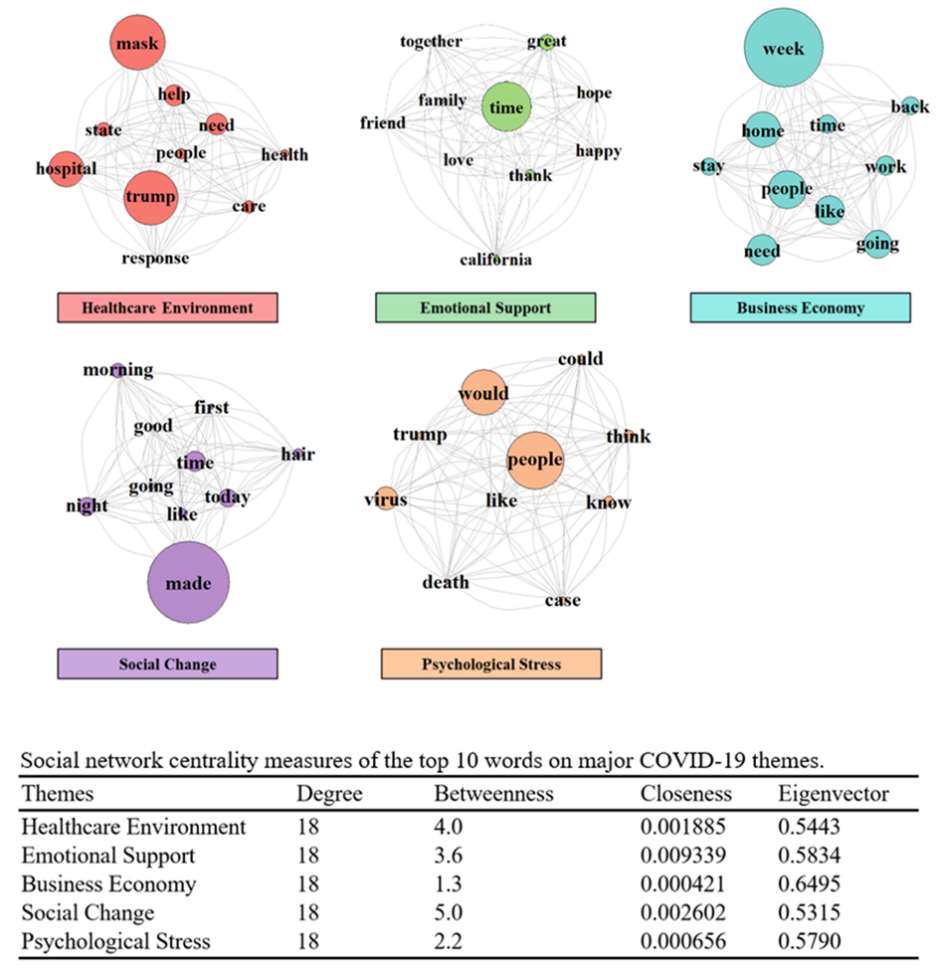
Social network graphs of the dominant topics about coronavirus disease (COVID-19), with the top 10 associated words per topic. The size of the node is proportional to the weight of the edges.

Topic modeling identified 5 salient topics that dominated Twitter discussions of COVID-19 and each of the 5 topics was labeled with a theme: health care environment [21], emotional support, business economy, social change, and psychological stress. Figure 4 displays 5 social network graphs, each corresponding to 1 of the 5 themes. Each social network graph shows the top 10 most frequently used words in tweets corresponding to a specific theme. The words are referred to as nodes or social actors when describing social network graphs, in which the size of the node represents the frequency of a certain word showing up. The lines between the words are referred to as links or actions, and these show the relationship between nodes. “Trump,” “mask,” and “hospital” dominated the discussion of health care environment. “Time” dominated the discussion of emotional support. “Week,” “people,” “home,” “work,” and “need” dominated business economy. For social change, “made,” “today,” and “time” dominated. In psychological stress, “people,” “would,” and “virus” dominated. Of note, Figure 4 reveals that among all 5 topics, the closeness centrality measure is the highest for emotional support, indicating that emotional support is the topic that is likely activated in each of the topic discussions.

**Figure 5:**
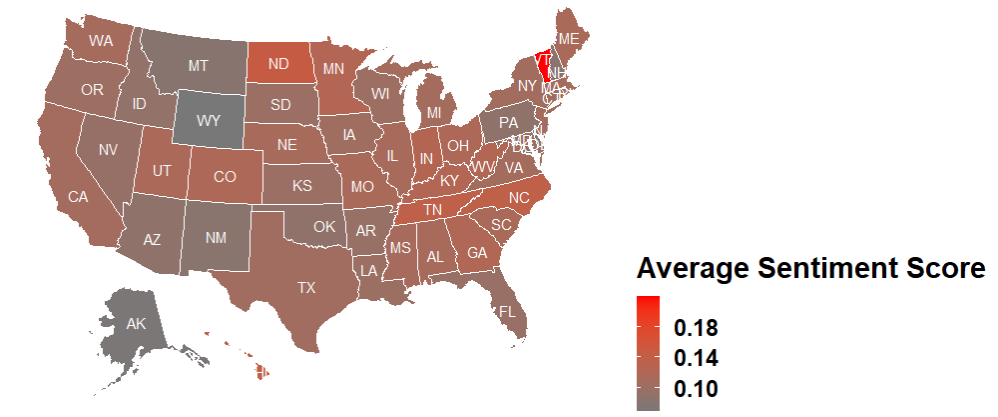
Heat map of the average sentiment score by state in the United States. A larger number represents a more positive sentiment score.

Figure 5 displays a heat map of the average sentiment score seen in each state in the United States. The darker the color of a certain state, the more negative the sentiment. Conversely, the lighter the color, the more positive the sentiment. Among the 50 states, Alaska, Wyoming, New Mexico, Pennsylvania, and Florida showed the most negative sentiment. Tweets from Vermont, North Dakota, Utah, Colorado, Tennessee, and North Carolina had the most positive sentiment in general.

## Discussion

### Overview

An unprecedented situation such as the COVID-19 outbreak makes it important to rapidly define the zeitgeist as new issues arise in a difficult time. This study found that overall, positive sentiment outweighed negative sentiment. Negative sentiment about COVID-19 was more prevalent in sparsely populated states with lower infection rates. Common negative sentiment tweet key words included “Trump,” “crisis,” “need,” “people,” “time,” “virus,” and “right.” Positive sentiment included words of encouragement and key phrases calling for the population to come together. These words included “thank,” “love,” “today,” “good,” and “friend.” The 5 most common themes were health care environment, business economy, emotional support, social change, and psychological stress, which represent the biggest concerns for the public.

### Sentiments

Sentiment analysis is useful to capture public perception of an event. Overall, sentiments expressed in tweets about the pandemic were more likely to be positive, implying that the public remained hopeful in the face of an unprecedented public health crisis. Positive sentiment keywords commonly expressed gratitude for frontline workers and community efforts to support vulnerable members of the community, but a few keywords conveyed negative sentiments toward those on the frontline. Some words drew a similarity to frontline workers and soldiers fighting a war. Another form of positive sentiment was the encouragement of infection prevention to maintain public health standards, such as the “Stay Home” trend. Overall, positive sentiments outweighed negative sentiments. The high proportion of positive sentiments suggests that some people might have underestimated the severity of COVID-19 during the early period of the pandemic. One cautionary note is that tweets generated in states experiencing lower rates of infection tended to be most positive, suggesting that states more directly impacted by the pandemic were more likely to be negative. Strategies targeted to high-impact areas may be needed to keep the public engaged and hopeful about their futures.

The negative sentiment keywords suggest that tweets may be a way to vent negative feelings about consequences imposed by COVID-19 restrictions. Negative keywords commonly featured “know” and “think,” words that pertain to information and information sharing. Information sharing can play into the public’s risk perception, which can be affected by an individual’s trust in authorities and (in)ability to recognize misinformation [22]. A recent study found that most Americans trust the director of the CDC or National Institutes of Health to lead the COVID-19 response over Congress [23]. This study also found that the public generally supports infection prevention measures in the United States.

### Twitter Discussion Themes Related to COVID-19

#### Health Care Environment

The discussions around the health care environment overlapped with politics. Vital supplies such as personal protective equipment and intensive care resources were linked to the need for government support. Health care workers discussed safety on the frontlines as an issue which negatively impacted their mental and physical health [24]. These findings tended to correlate to health care environments in COVID-19 hotspots affected by massive redistributions of resources [25].

#### Business Economy

A particularly stressful part of the shutdown has been its impact on the US and global economy [26]. Millions of people lost jobs, and unemployment numbers rose rapidly. The term “week” was a significant topic and was linked with conversations about “work”: the words “going,” “back,” and “need” were mentioned. Many people were unemployed or worked from home, and a common topic was “home,” “work,” and “stay” interlinked with the term “like.” These findings indicate that while discussions overwhelmingly centered around the week and work concerns, not all conversations related to loss of work. Some tweets indicated liking working from home. This finding suggests that strategies to reopen the economy that embrace working from home may both be popular and also help to reduce SARS-CoV-2 spread.

#### Emotional Support

Support from one’s network of family and friends during periods of high stress can help reduce its harmful effects [27,28]. However, social isolation and distancing can preclude receiving the support needed. Our results showed that “time” was an overwhelming topic of discussion and clearly linked to “family,” “friend,” “together,” and “hope.” It may be that despite isolation, individuals find comfort and support through social media connections with their family and friends, while remaining hopeful they will soon have time together.

#### Social Change

The pandemic and its associated societal upheaval appeared to leave individuals in a state of uncertainty. Overwhelmingly, discussions focused on the word “made,” with links to “today,” “night,” and “morning.” Changes made to individuals’ lives included daily activity restrictions, which could occur in different way across the entire day. Some changes could be “good,” or individuals may “like” some changes. An interesting finding was “hair.” Shutdown orders for nonessential businesses included hair salons, and these restrictions likely impact individuals’ self-perception of their appearance and the way they maintain their personal grooming.

#### Psychological Stress

Psychological stress can be acute or chronic and both physiologically and psychologically detrimental [29,30]. Acute stress can become chronic if one is repeatedly exposed to stressful events. The current pandemic took what could have been an acute stress (eg, having the flu) and transformed it into a chronic stressful situation of worldwide sickness and death, and economic disruption. Our study found discussions of psychological stress overlapped with politics. Most discussions centered around “people,” highly overlapping with “virus,” “case,” and “death.” “Would,” also featured prominently, along with “could” and “know,” suggesting that individuals were concerned with the impact that the virus might have on people they know. Discussion also connected “Trump” with “people,” “death,” and “virus,” suggesting that other discussions were focused on the role of the president in leading people to fight the virus and minimize death.

It is unclear why the number of tweets declined during the study period and followed a power law distribution as shown in Figure 1. One possible explanation is that most people tend to have more questions and discussions regarding a phenomenon when it is novel, but the discussions may slow down as time progresses. It is also noted that the behavior of extremely rare events such as stock market crashes and large natural disasters seem to follow the power law distribution [31]. Future research is needed to explore this fascinating pattern and understand the driving force behind the distribution.

The first case of COVID-19 in the United States was reported on January 19, 2020 [32]. By mid-March, all 50 states and 4 United States territories had reported cases. Of the 12,757 COVID-19–related reported deaths as of April 7, approximately half of all deaths were from New York and New Jersey with case-fatality ratios being lowest in Utah [26]. The more positive sentiment of Utah may be in part due to the lower incidences of reported cases and its having the lowest ratio of COVID-19 fatalities in April.

### Potential Impact

Our results demonstrate that applying ML methods to mine pandemic-related tweets can yield useful data for agencies, local leaders, and health providers. For example, this study found that sentiments differed by region and geocaching tweets can allow localities to leverage data to match strategies and communications to community needs. When properly analyzed, digital data such as tweets can add to real-time epidemiologic data [33], allowing a more comprehensive and instantaneous evaluation of the pandemic situation. This is important since traditional public health data may take 1 to 2 weeks to become available. By virtue of the sheer volume, Twitter data might also help to identify or track rare event occurrences such as the multisystem inflammatory syndrome associated with COVID-19 in children [16].

In addition, Twitter offers an inexpensive and efficient platform to evaluate the effectiveness of public health communications [34], and to target public health campaigns on the dominant topics of Twitter discussion. For example, tweet analysis regarding mask wearing and hand hygiene can assess messaging. Applying ML to tweets can also provide insight into how the public interprets mixed messages regarding therapies such as hydroxychloroquine.

As the likelihood of a new coronavirus vaccine increases, one concern is that despite the established value of vaccines, only about half the public might elect to take a coronavirus vaccine [35,36]. Even a clinically proven vaccine depends on a high level of acceptance [37] and unsubstantiated concerns about negative side effects might overshadow the benefits of coronavirus immunization. Twitter offers an opportunity to follow vaccine acceptance and to tailor responses to those who oppose vaccination. The local risk of vaccine-preventable diseases can rise when there is a geographic aggregation of persons refusing vaccination and expressing more negative sentiments. Twitter analysis provides a potentially powerful and inexpensive tool for public health officials to identity geographic clusters for interventions and to evaluate their effectiveness.

### Limitations

One limitation is that Twitter represents community interaction and its user profiles contain little demographic data, rendering an analysis of Twitter user demographic subgroups meaningless. An analysis of subgroups might yield more insights. Further, Twitter users do not fully represent the United States population, since only 15% of adults use Twitter, and younger adults aged 18 to 29 years old and minorities tend to be more active in Twitter discussions than the general population [38]. Additionally, active and passive Twitter users are more prevalent than moderate users [38]. With such potentially nonuniform sampling distribution and nonrepresentative demographic distribution of the United States population, the findings of specific sentiments can be biased [39], thus cautious interpretation of the findings is needed. However, the number of Twitter users over age 65 continues to increase, reducing the level of age-related bias. Additionally, the overrepresentation of minorities may be a strength in terms of assessing health disparities.

The real-time posting of tweets is both a strength and a weakness. A strength is that it captures what is happening at the time, but a weakness is that tweet content can evolve very quickly [40], thus requiring constant monitoring of posts. In addition, the use of Twitter is not uniform across time or geography. Padilla et al [41] noted that Thursdays and Saturdays have slightly higher sentiment scores, but this study did not factor such differences into our analyses. Nevertheless, our results illustrate the insights that monitoring tweets can provide to a health-related event. Using ML to assess tweets is a potential weakness since it may not perform as well as human curation [42]. However, a strength is that ML processes a vast amount of data much faster than human methods. Finally, while social media may not capture the sentiment of those less vocal, a tweet analysis can provide insight into the type of information they process.

### Conclusions

This study identified 5 overarching themes related to COVID-19: health care environment, emotional support, business economy, social change, and psychological stress. “Trump,” “mask,” and “hospital” dominated the tweets of health care environment. “Week,” “people,” “home,” “work,” and “need” dominated business economy. In psychological stress, “people,” “would,” and “virus” dominated the discussion. Overall, positive tweets outweighed negative tweets. The sentiments can clarify the public response to COVID-19 and help guide government officials, private entities, and the public with information as they navigate the pandemic.

## Appendix

Multimedia Appendix 1 Computer code for data collection, data analyses, and figure generation.

## Abbreviations

COVID-19: coronavirus disease
CDC: Centers for Disease Control and Prevention
LDA: Latent Dirichlet Allocation
ML: machine learning
PTSD: posttraumatic stress disorder
SARS-CoV-2: severe acute respiratory syndrome coronavirus 2
VADER: Valence Aware Dictionary and sEntiment Reasoner
WHO: World Health Organization
