# Supplementary material for: Social Network Analysis of COVID-19 Sentiments: Application of Artificial Intelligence
Source: J Med Internet Res. 2020 Aug 18;22(8):e22590. doi: 10.2196/22590 (PMC7438102; doi:10.2196/22590)
Supplement: Multimedia Appendix 1 [file jmir_v22i8e22590_app1.docx]

Appendix 1: Computer code for data collection, data analyses and figure generations.

| Task | Computer Code |
| --- | --- |
| Scraping twitter data from twint (49 US states excluding HI and AK; Data obtained in 2-hour intervals due to volume. There are 12 files per day) | all keywords twint.ipynb  import twint import nest_asyncio nest_asyncio.apply()  import pandas as pd  c = twint.Config()  c.Search = "coronavirus OR covid OR COVID-19 OR SARS-CoV-2 OR stayhome OR covid19 OR lockdown OR \"shelter in place\" OR coronavirustruth OR outbreak OR pandemic OR quarantine OR \"social distancing\" OR hoax OR \"be kind\" OR \"health heroes\" OR ppe OR isolation OR \"school cancelled\" OR \"online teaching\" OR homeschooling" c.Show_hashtags = True  #time period of data scraped (data is scraped in 2 hour batches from 03/20/2020 00:00:00 to 04/19/2020 23:59:59)  c.Since = '2020-04-19 00:00:00' c.Until = '2020-04-19 01:59:59'  c.Near = "USA" c.Lang = "en" c.Hide_output = True  #c.Limit = 20  c.Location = True  c.Store_csv = True c.Output = 'apr19_1.csv'  #run the search twint.run.Search(c) |
| Scraping twitter data from twint (HI) (Data obtained in daily intervals) | Twint Hawaii.ipynb  import twint  import pandas as pd  c = twint.Config()  c.Search = "coronavirus OR covid OR COVID-19 OR SARS-CoV-2 OR stayhome OR covid19 OR lockdown OR \"shelter in place\" OR coronavirustruth OR outbreak OR pandemic OR quarantine OR \"social distancing\" OR hoax OR \"be kind\" OR \"health heroes\" OR ppe OR isolation OR \"school cancelled\" OR \"online teaching\" OR homeschooling" c.Show_hashtags = True  c.Since = '2020-04-18' c.Until = '2020-04-19'  #c.Near = "Honolulu" #get tweets within 1000km radius from Honolulu c.Geo = "21.315603,-157.858093,1000km" c.Lang = "en" c.Hide_output = True  #c.Limit = 20  c.Location = True  c.Store_csv = True c.Output = 'apr18_HI.csv'  #run the search twint.run.Search(c)  from google.colab import files files.download('apr18_HI.csv') |
| Scraping twitter data from twint (AK) (Data obtained in daily intervals) | Twint Alaska.ipynb  import twint  import pandas as pd  c = twint.Config()  c.Search = "coronavirus OR covid OR COVID-19 OR SARS-CoV-2 OR stayhome OR covid19 OR lockdown OR \"shelter in place\" OR coronavirustruth OR outbreak OR pandemic OR quarantine OR \"social distancing\" OR hoax OR \"be kind\" OR \"health heroes\" OR ppe OR isolation OR \"school cancelled\" OR \"online teaching\" OR homeschooling" c.Show_hashtags = True  c.Since = '2020-04-19' c.Until = '2020-04-20'  #get tweets within 1000km radius from Fairbanks, Alaska c.Geo = "64.835365,-147.776749,3000km" c.Lang = "en" c.Hide_output = True  #c.Limit = 20  c.Location = True  c.Store_csv = True c.Output = 'apr19_AK.csv'  #run the search twint.run.Search(c)  from google.colab import files files.download('apr19_AK.csv') |
| Join the 12 files of the bi-hourly data from the 49 states to produce one daily file for the 49 states | concatenate dataframe.ipynb  import pandas as pd  #concatenate the dataframe to output complete data for one day df1 = pd.read_csv("apr2_1.csv") df2 = pd.read_csv("apr2_2.csv") df3 = pd.read_csv("apr2_3.csv") df4 = pd.read_csv("apr2_4.csv") df5 = pd.read_csv("apr2_5.csv") df6 = pd.read_csv("apr2_6.csv") df7 = pd.read_csv("apr2_7.csv") df8 = pd.read_csv("apr2_8.csv") df9 = pd.read_csv("apr2_9.csv") df10 = pd.read_csv("apr2_10.csv") df11 = pd.read_csv("apr2_11.csv") df12 = pd.read_csv("apr2_12.csv")  frames = [df12, df11, df10, df9, df8, df7, df6, df5, df4, df3, df2, df1] df = pd.concat(frames) df = df.reset_index(drop =True)  df.to_csv("apr2.csv", index = False) |
| Merge Hawaii and Alaska into 49 states daily data + data cleaning | Clean data and include Hawaii and Alaska.ipynb  import pandas as pd  *#48 states + DC file* df49 = pd.read_csv("Data files/apr19.csv") *#Hawaii file* dfhi = pd.read_csv("Hawaii/apr19_HI.csv") *#Alaska file* dfak = pd.read_csv("Alaska/apr19_AK.csv")  *#separate city and state into different columns for dfhi* new = dfhi["place"].str.split(",", n = 1, expand = True)  dfhi["city"]= new[0]  dfhi["state"]= new[1]  dfhi.drop(columns =["place"], inplace = True)   *#remove NAs in state column* dfhi = dfhi.dropna(subset=["state"]) *#remove whitespaces in state column* dfhi["state"] = dfhi["state"].str.strip() *#subset by state that are properly formatted only* dfhi = dfhi[dfhi["state"].str.len() < 3] dfhi = dfhi[dfhi["state"].str.contains("HI")]  dfhi[["date", "time", "username", "tweet", "hashtags", "likes_count", "replies_count", "retweets_count", "city", "state", "mentions"]]  *#separate city and state into different columns for dfak* new = dfak["place"].str.split(",", n = 1, expand = True)  dfak["city"]= new[0]  dfak["state"]= new[1]  dfak.drop(columns =["place"], inplace = True)   *#remove NAs in state column* dfak = dfak.dropna(subset=["state"]) *#remove whitespaces in state column* dfak["state"] = dfak["state"].str.strip() *#subset by state that are properly formatted only* dfak = dfak[dfak["state"].str.len() < 3] dfak = dfak[dfak["state"].str.contains("AK")]  dfak[["date", "time", "username", "tweet", "hashtags", "likes_count", "replies_count", "retweets_count", "city", "state", "mentions"]]  *#separate city and state into different columns for df49* new = df49["place"].str.split(",", n = 1, expand = True)  df49["city"]= new[0]  df49["state"]= new[1]  df49.drop(columns =["place"], inplace = True)   *#remove NAs in state column* df49 = df49.dropna(subset=["state"]) *#remove whitespaces in state column* df49["state"] =df49["state"].str.strip() *#subset by state that are properly formatted only* df49 = df49[df49["state"].str.len() < 3] df49 = df49[df49["state"].str.contains("AL\|AK\|AZ\|AR\|CA\|CO\|CT\|DC\|DE\|FL\|GA\|HI\|ID\|IL\|IN\|IA\|KS\|KY\|LA\|ME\|MD\|MA\|MI\|MN\|MS\|MO\|MT\|NE\|NV\|NH\|NJ\|NM\|NY\|NC\|ND\|OH\|OK\|OR\|PA\|RI\|SC\|SD\|TN\|TX\|UT\|VT\|VA\|WA\|WV\|WI\|WY")]  df51 = pd.concat([df49,dfhi,dfak]) df51[["date", "time", "username", "tweet", "hashtags", "likes_count", "replies_count", "retweets_count",  *"city"*, "state", "mentions"]]  df51.to_csv("complete data/apr19_all.csv", index = False)  df51 = pd.read_csv("complete data/apr19_all.csv") df51[["date", "time", "username", "tweet", "hashtags", "likes_count", "replies_count", "retweets_count", "city", "state", "mentions"]] |
| Combine all 31 days of data into one file + data cleaning | Data cleaning no sampling.ipynb  import pandas as pd  #read all the csv files to data frames (will update with more files later) df1 = pd.read_csv("complete data/mar20_all.csv") df2 = pd.read_csv("complete data/mar21_all.csv") df3 = pd.read_csv("complete data/mar22_all.csv") df4 = pd.read_csv("complete data/mar23_all.csv") df5 = pd.read_csv("complete data/mar24_all.csv") df6 = pd.read_csv("complete data/mar25_all.csv") df7 = pd.read_csv("complete data/mar26_all.csv") df8 = pd.read_csv("complete data/mar27_all.csv") df9 = pd.read_csv("complete data/mar28_all.csv") df10 = pd.read_csv("complete data/mar29_all.csv") df11 = pd.read_csv("complete data/mar30_all.csv") df12 = pd.read_csv("complete data/mar31_all.csv") df13 = pd.read_csv("complete data/apr1_all.csv") df14 = pd.read_csv("complete data/apr2_all.csv") df15 = pd.read_csv("complete data/apr3_all.csv") df16 = pd.read_csv("complete data/apr4_all.csv") df17 = pd.read_csv("complete data/apr5_all.csv") df18 = pd.read_csv("complete data/apr6_all.csv") df19 = pd.read_csv("complete data/apr7_all.csv") df20 = pd.read_csv("complete data/apr8_all.csv") df21 = pd.read_csv("complete data/apr9_all.csv") df22 = pd.read_csv("complete data/apr10_all.csv") df23 = pd.read_csv("complete data/apr11_all.csv") df24 = pd.read_csv("complete data/apr12_all.csv") df25 = pd.read_csv("complete data/apr13_all.csv") df26 = pd.read_csv("complete data/apr14_all.csv") df27 = pd.read_csv("complete data/apr15_all.csv") df28 = pd.read_csv("complete data/apr16_all.csv") df29 = pd.read_csv("complete data/apr17_all.csv") df30 = pd.read_csv("complete data/apr18_all.csv") df31 = pd.read_csv("complete data/apr19_all.csv")  frames = [df1, df2, df3, df4, df5, df6, df7, df8, df9, df10,   df11, df12, df13, df14, df15, df16, df17, df18, df19, df20,  df21, df22, df23, df24, df25, df26, df27, df28, df29, df30, df31] df = pd.concat(frames) df = df.reset_index(drop =True)  #subset the data frame by the following columns df_clean = df[["date", "username", "tweet", "hashtags", "likes_count", "replies_count", "retweets_count", "city"*,* "state", "mentions"]]  #drop any rows containing NA  df_clean = df_clean.dropna()  #convert columns to the correct data types df_clean = df_clean.astype({"likes_count": int, "replies_count": int, "retweets_count": int})  df_clean["place"] = df_clean["city"] + ' ' + df_clean["state"] df_clean.to_csv("complete data no sampling all.csv", index = False)  #convert certain columns to lowercase df_clean['tweet'] = df_clean['tweet'].str.lower() df_clean['hashtags'] = df_clean['hashtags'].str.lower()  #df_clean['likes_count'].sum() df_clean['retweets_count'].sum()  df_mentions = df_clean[["mentions"]]  import ast df_mentions['mentions'] = df_mentions['mentions'].apply(ast.literal_eval)  df_mentions.to_csv("df_mentions_alldat.csv")  df_clean.username.nunique()  #get frequency counts of keywords  df_clean.tweet.str.count("online teaching").sum()  #get the number of unique values in a column df_clean.state.nunique()  #get the number of unique cities df_clean["place"] = df_clean["city"] + ' ' + df_clean["state"] df_clean.place.nunique() |
| Tweets cleaning, sentiment analysis with Vader, word cloud generation | vader sentiment analysis with wordcloud - overall data.ipynb Tweets Cleaning import pandas as pd import re import numpy as np import nltk import string import warnings  samp_df = pd.read_csv("complete data no sampling all.csv")  samp_df = samp_df.reset_index(drop=True)  samp_df.tail()  #function to remove patterns def remove_pattern(input_txt, pattern):  r = re.findall(pattern, input_txt)  for i in r:  input_txt = re.sub(i, '', input_txt)    return input_txt  # remove twitter handles (@user) samp_df['tidy_tweet'] = np.vectorize(remove_pattern)(samp_df['tweet'], "@[\w]*")  #remove urls samp_df['tidy_tweet'] = samp_df['tidy_tweet'].str.replace('http\S+\|www.\S+\|pic.\S+', '', case=False)  #remove hashtags samp_df['tidy_tweet'] = samp_df['tidy_tweet'].str.replace("#", "")  #remove numbers samp_df['tidy_tweet'] = samp_df['tidy_tweet'].str.replace('\d+', '')  #remove extra whitespace samp_df['tidy_tweet'] = samp_df['tidy_tweet'].str.replace('\s+', ' ')  #combine keywords that are multiple words into one word (for stopwords because stopwords cannot handle multi-word phrases) samp_df['tidy_tweet'] = samp_df['tidy_tweet'].str.replace("social distancing", "socialdistancing") samp_df['tidy_tweet'] = samp_df['tidy_tweet'].str.replace("shelter in place", "shelterinplace") samp_df['tidy_tweet'] = samp_df['tidy_tweet'].str.replace("be kind", "bekind") samp_df['tidy_tweet'] = samp_df['tidy_tweet'].str.replace("health heroes", "healthheroes") samp_df['tidy_tweet'] = samp_df['tidy_tweet'].str.replace("school cancelled", "schoolcancelled") samp_df['tidy_tweet'] = samp_df['tidy_tweet'].str.replace("online teaching", "onlineteaching")  #remove stopwords from nltk.corpus import stopwords  stop = stopwords.words('english')   #extra stopwords for sentiment analysis add_words = ["coronavirus", "covid", "COVID-19", "SARS-CoV-2", "stayhome", "covid19", "lockdown", "shelterinplace",  "coronavirustruth", "outbreak", "pandemic", "quarantine", "socialdistancing", "hoax", "bekind",  "healthheroes", "ppe", "isolation", "homeschooling", "schoolcancelled", "onlineteaching", "covid_"] stop.extend(add_words)  #more extra stopwords add_words = ["st", "th", "nd", "rd", "..."] stop.extend(add_words)  #exclude negations from list of stopwords remove_words = {"not", "isn't", "aren't", "wasn't", "weren't", "hasn't", "haven't", "hadn't", "doesn't", "don't", "didn't",   "won't", "wouldn't", "shan't", "shouldn't", "can't", "cannot", "couldn't", "mustn't"}   stop = set([word for word in stop if word not in remove_words])  samp_df['tidy_tweet'] = samp_df['tidy_tweet'].apply(lambda x: ' '.join([word for word in x.split() if word not in (stop)])) Lemmatize tweets #tokenize tweets tokenized_tweet = samp_df['tidy_tweet'].apply(lambda x: x.split()) tokenized_tweet.head()  #nltk.download('wordnet')  #lemmatize tweets from nltk.stem import WordNetLemmatizer  lmtzr = nltk.WordNetLemmatizer()  tokenized_tweet = tokenized_tweet.apply(lambda x: [lmtzr.lemmatize(i) for i in x]) tokenized_tweet.head()  for i in range(len(tokenized_tweet)):  tokenized_tweet[i] = ' '.join(tokenized_tweet[i])  samp_df['tidy_tweet'] = tokenized_tweet  samp_df["tweet"][35]  samp_df["tidy_tweet"][32]  samp_df.to_csv("clean data vader.csv", index = False) Sentiment Analysis import pandas as pd import matplotlib.pyplot as plt import seaborn as sns from vaderSentiment.vaderSentiment import SentimentIntensityAnalyzer analyser = SentimentIntensityAnalyzer()  df = pd.read_csv("clean data vader.csv")  df = df.dropna()  def get_score(text):  score = analyser.polarity_scores(text)  return score  df["score"] = df['tidy_tweet'].apply(get_score)  df = pd.concat([df.drop(['score'], axis=1), df['score'].apply(pd.Series)], axis=1)  df.rename({'neu': 'neutral', 'pos': 'positive', 'neg': 'negative', 'compound': 'compound_score'}, axis=1, inplace=True)  def get_tweet_sentiment(text):  if text >= 0.05:  return 'positive'  elif (text > -0.05) and (text < 0.05):  return 'neutral'  else:  return 'negative'  df["sentiment"] = df["compound_score"].apply(get_tweet_sentiment)  df["sentiment"].value_counts()  pd.set_option('display.max_colwidth', 0) df[["tweet", "tidy_tweet", "compound_score", "sentiment"]]  df["tweet"][5]  df.to_csv("clean data vader with sentiment with polarity.csv", index = False) WordCloud from wordcloud import WordCloud, STOPWORDS, ImageColorGenerator import pandas as pd import re import numpy as np import nltk import string import warnings import matplotlib.pyplot as plt  df = pd.read_csv('clean data vader with sentiment with polarity.csv') df['tidy_tweet'] = df['tidy_tweet'].str.lower()  df.head()  #remove all special characters (leaving only letters) df['tidy_tweet'] = df['tidy_tweet'].str.replace("[^a-zA-Z]", ' ') #remove extra whitespace df['tidy_tweet'] = df['tidy_tweet'].str.replace('\s+', ' ') #remove words with less than 4 letters df['tidy_tweet'] = df['tidy_tweet'].apply(lambda x: ' '.join([w for w in x.split() if len(w)>3]))  #combine keywords that are multiple words into one word (for stopwords because stopwords cannot handle multiple phrases) df['tidy_tweet'] = df['tidy_tweet'].str.replace("social distancing", "socialdistancing") df['tidy_tweet'] = df['tidy_tweet'].str.replace("shelter in place", "shelterinplace") df['tidy_tweet'] = df['tidy_tweet'].str.replace("be kind", "bekind") df['tidy_tweet'] = df['tidy_tweet'].str.replace("health heroes", "healthheroes") df['tidy_tweet'] = df['tidy_tweet'].str.replace("school cancelled", "schoolcancelled") df['tidy_tweet'] = df['tidy_tweet'].str.replace("online teaching", "onlineteaching")  from nltk.corpus import stopwords  stop = stopwords.words('english')  add_words = ["coronavirus", "covid", "COVID-19", "SARS-CoV-2", "stayhome", "covid19", "lockdown", "shelterinplace",  "coronavirustruth", "outbreak", "pandemic", "quarantine", "socialdistancing", "hoax", "bekind",  "healthheroes", "ppe", "isolation", "homeschooling", "schoolcancelled", "onlineteaching", "via"] stop.extend(add_words)  df['tidy_tweet'] = df['tidy_tweet'].apply(lambda x: ' '.join([word for word in x.split() if word not in (stop)]))  df = df.dropna()  df.to_csv("clean data vader with sentiment wordcloud with polarity.csv", index = False)  df.head()  positive = df[df["sentiment"]=="positive"] negative = df[df["sentiment"]=="negative"] neutral = df[df["sentiment"]=="neutral"]  def word_cloud(wd_list):  all_words = ' '.join([text for text in wd_list])  wordcloud = WordCloud(  background_color='white',  stopwords=list(STOPWORDS),  width=1600,  height=800,  random_state=21,  colormap='jet',  max_words=50,  max_font_size=200).generate(all_words)  plt.figure(figsize=(12, 10))  plt.axis('off')  plt.imshow(wordcloud, interpolation="bilinear"); Word Cloud for all tweets #word cloud that does not separate by sentiments word_cloud(df["tidy_tweet"]) Word cloud for positive sentiment word_cloud(positive["tidy_tweet"]) Word cloud for negative sentiment word_cloud(negative["tidy_tweet"]) Word cloud for neutral sentiment word_cloud(neutral["tidy_tweet"]) |
| Topic modeling using LDA | topic modeling with sentiment scores.ipynb  from google.colab import drive  drive.mount('/content/gdrive')  import pandas as pd import numpy as np  import matplotlib.pyplot as plt import seaborn as sns  import sklearn  import re import nltk import string  df = pd.read_csv('gdrive/My Drive/corona/Data/clean data vader with sentiment wordcloud with polarity.csv')  #shuffle the data df = df.sample(frac = 1, random_state = 1) df = df.reset_index(drop=True)  df.tail() Obtaining themes using topic modeling methods df = df.dropna() from nltk.corpus import stopwords nltk.download('stopwords') stop = stopwords.words('english') df['tidy_tweet'] = df['tidy_tweet'].apply(lambda x: ' '.join([word for word in x.split() if word not in (stop)]))  df.tail()  from sklearn.feature_extraction.text import CountVectorizer, TfidfTransformer from sklearn.preprocessing import normalize # the vectorizer object will be used to transform text to vector form vectorizer = CountVectorizer(max_df=0.9, min_df=100, token_pattern='\w+\|\$[\d\.]+\|\S+') # apply transformation tf = vectorizer.fit_transform(df['tidy_tweet']) #.toarray() # tf_feature_names tells us what word each column in the matrix represents tf_feature_names = vectorizer.get_feature_names() tf.shape Topic Modeling LDA from sklearn.decomposition import LatentDirichletAllocation  def display_topics(model, feature_names, no_top_words):  topic_dict = {}  for topic_idx, topic in enumerate(model.components_):  topic_dict["Topic %d words" % (topic_idx)]= ['{}'.format(feature_names[i])  for i in topic.argsort()[:-no_top_words - 1:-1]]  topic_dict["Topic %d weights" % (topic_idx)]= ['{:.1f}'.format(topic[i])  for i in topic.argsort()[:-no_top_words - 1:-1]]  return pd.DataFrame(topic_dict)  model = LatentDirichletAllocation(n_components=5, random_state=45)  # fit data to model model.fit(tf) no_top_words = 10  display_topics(model, tf_feature_names, no_top_words)  def display_topics2(model, feature_names, no_top_words):  topic_dict = {}  for topic_idx, topic in enumerate(model.components_):  topic_dict["Topic %d words" % (topic_idx)]= ['{}'.format(feature_names[i])  for i in topic.argsort()[:-no_top_words - 1:-1]]  return pd.DataFrame(topic_dict)  topics_df = display_topics2(model, tf_feature_names, no_top_words) topics_df  # create topic matrix lda_output = model.fit_transform(tf)  # column names topicnames = topics_df.columns  # index names docnames = ["Doc" + str(i) for i in range(len(df))]  # Make the pandas dataframe df_document_topic = pd.DataFrame(np.round(lda_output, 2), columns=topicnames, index=docnames)  # Get dominant topic for each document dominant_topic = np.argmax(df_document_topic.values, axis=1) df_document_topic['dominant_topic'] = dominant_topic df_document_topic['percentage_contribution'] = df_document_topic[['Topic 0 words', 'Topic 1 words', 'Topic 2 words', 'Topic 3 words', 'Topic 4 words']].values.max(1)  df_document_topic.reset_index(inplace=True) df_sent_topic= pd.merge(df, df_document_topic, left_index=True, right_index=True) df_sent_topic.drop('index', axis=1, inplace=True)  df_document_topic['percentage_contribution'] = df_document_topic[['Topic 0 words', 'Topic 1 words', 'Topic 2 words', 'Topic 3 words', 'Topic 4 words']].values.max(1)  df_document_topic.reset_index(inplace=True) df_sent_topic= pd.merge(df, df_document_topic, left_index=True, right_index=True) df_sent_topic.drop('index', axis=1, inplace=True)  df_sent_topic.tail()  df_sent_topic.to_csv("gdrive/My Drive/corona/Data/dominant topic scores all lda with polarity.csv") |
| Analysis and graphs | analysis and graphs.ipynb  from google.colab import drive  drive.mount('/content/gdrive')  import pandas as pd import numpy as np import matplotlib.pyplot as plt import seaborn as sns import sklearn import re import nltk import string  from statistics import stdev  df = pd.read_csv('gdrive/My Drive/corona/Data/dominant topic scores all lda with polarity.csv', index_col=[0])  df = df[~df.date.str.contains("\[")] df['date'] = pd.to_datetime(df['date']) df = df.sort_values(by='date')  df = df.reset_index(drop=True) df.tail()  count = df['tidy_tweet'].str.split().str.len() df = df[~(count<3)]  df['date_ordinal'] = pd.to_datetime(df['date']).apply(lambda date: date.toordinal())  df.tail() Counts (for paper) df.info()  df.username.nunique()  df.state.nunique()  df.place.nunique()  df['retweets_count'].dropna().astype(int).sum()  import ast df_mentions = df[["mentions"]] df_mentions['mentions'] = df_mentions['mentions'].dropna().apply(ast.literal_eval) df_mentions = df_mentions.explode('mentions') df_mentions = df_mentions.dropna() df_mentions  df_mentions.info()  df.sentiment.value_counts() Analysis from statistics import median from statistics import mode from scipy.stats import iqr  topic = df[(df.sentiment == "neutral") & (df.dominant_topic == 4)] #topic = df[(df["dominant_topic"] == 4)] compound_score = topic["compound_score"] print("mean = " + str(np.mean(compound_score))) print("SD = " + str(stdev(compound_score))) print("min = " + str(min(compound_score))) print("max = " + str(max(compound_score))) print("median = " + str(median(compound_score))) print("mode = " + str(mode(compound_score))) print("IQR = " + str(iqr(compound_score)))  topic.head()  len(df[(df.compound_score == 0.0) & (df.dominant_topic == 0)])  len(df[df.dominant_topic == 0]) Cluster bar graph topic0 = df[(df["dominant_topic"] == 0)] #create a list of sentiment values sorted alphabetically topic0_list = list(topic0.sentiment.value_counts().sort_index()) topic0_list  topic0_percent = [x/sum(topic0_list)*100 for x in topic0_list] topic0_percent = [ round(elem, 1) for elem in topic0_percent] topic0_percent  topic1 = df[(df["dominant_topic"] == 1)] topic1_list = list(topic1.sentiment.value_counts().sort_index()) topic1_list  topic1_percent = [x/sum(topic1_list)*100 for x in topic1_list] topic1_percent = [ round(elem, 1) for elem in topic1_percent] topic1_percent  topic2 = df[(df["dominant_topic"] == 2)] topic2_list = list(topic2.sentiment.value_counts().sort_index()) topic2_list  topic2_percent = [x/sum(topic2_list)*100 for x in topic2_list] topic2_percent = [ round(elem, 1) for elem in topic2_percent] topic2_percent  topic3 = df[(df["dominant_topic"] == 3)] topic3_list = list(topic3.sentiment.value_counts().sort_index()) topic3_list  topic3_percent = [x/sum(topic3_list)*100 for x in topic3_list] topic3_percent = [ round(elem, 1) for elem in topic3_percent] topic3_percent  topic4 = df[(df["dominant_topic"] == 4)] topic4_list = list(topic4.sentiment.value_counts().sort_index()) topic4_list  topic4_percent = [x/sum(topic4_list)*100 for x in topic4_list] topic4_percent = [ round(elem, 1) for elem in topic4_percent] topic4_percent  topic1.sentiment.value_counts()  topic1_list  topics_list = [] topics_list.append(topic0_list) topics_list.append(topic1_list) topics_list.append(topic2_list) topics_list.append(topic3_list) topics_list.append(topic4_list) topics_list  negative = [item[0] for item in topics_list] neutral = [item[1] for item in topics_list] positive = [item[2] for item in topics_list]  positive  percents_list = [] percents_list.append(topic0_percent) percents_list.append(topic1_percent) percents_list.append(topic2_percent) percents_list.append(topic3_percent) percents_list.append(topic4_percent) percents_list  negative_percent = [str(item[0]) + '%' for item in percents_list] neutral_percent = [str(item[1]) + '%' for item in percents_list] positive_percent = [str(item[2]) + '%' for item in percents_list]  negative_percent  from pylab import rcParams font = {'family' : 'normal',  'weight' : 'bold',  'size' : 16}  plt.rc('font', **font) rcParams['figure.figsize'] = 12, 8  positive  #this is the correct bar chart import numpy as np import matplotlib.pyplot as plt from matplotlib import rc import pandas as pd   # y-axis in bold rc('font', weight='bold')   # Values of each group bars1 = positive bars2 = neutral bars3 = negative   # Heights of bars1 + bars2 bars = np.add(bars1, bars2).tolist()   # The position of the bars on the x-axis r = [0,1,2,3,4]   # Names of group and bar width names = ['Healthcare\nEnvironment', 'Emotional\nSupport', 'Business\nEconomy', 'Social\nChange', 'Psychological\nStress'] barWidth = 0.5  fig, ax = plt.subplots() # Create brown bars plt.bar(r, bars1, color='#006D77', width=barWidth, label = 'positive') # Create green bars (middle), on top of the firs ones plt.bar(r, bars2, bottom=bars1, color='#83C5BE', width=barWidth, label = 'neutral') # Create green bars (top) plt.bar(r, bars3, bottom=bars, color='#E29578', width=barWidth, label = 'negative')   # Custom X axis plt.xticks(r, names, fontweight='bold') plt.xlabel('Dominant Topic', fontweight='bold') plt.ylabel('Number of Tweets', fontweight='bold') plt.legend()  import matplotlib as mpl ax.yaxis.set_major_formatter(mpl.ticker.StrMethodFormatter('{x:,.0f}'))  # Show graphic plt.savefig("gdrive/My Drive/corona/Data/stacked bargraph correct.png") plt.show() Heat map of United States Produce a csv file for the heatmap. Heatmap is generated in R under the file 'state_heatmap for paper revised.R'  state_df = df.groupby(['state'])['compound_score'].mean().reset_index() state_df.head() state_df.to_csv("state_df3.csv")  from google.colab import files files.download('state_df3.csv') Number of tweets and sentiments from datetime import date  density_df = df[["date", "sentiment", "date_ordinal"]] density_df.tail()  from pylab import rcParams font = {'family' : 'normal',  'weight' : 'bold',  'size' : 20}  plt.rc('font', **font) rcParams['figure.figsize'] = 15,10  import matplotlib.pyplot as plt import seaborn as sns fig, ax = plt.subplots() import matplotlib.dates as mdates  fig.autofmt_xdate() # matplotlib histogram plt.hist(density_df['date'], color = 'black', edgecolor = 'white',  bins = int(31), rwidth=0.8)  plt.xlabel('Date', fontsize=24, fontweight= "bold") plt.ylabel('Number of Tweets', fontsize=24, fontweight= "bold") #plt.savefig('drive/My Drive/corona/Data/numberoftweets.png') Mentions count (misc for paper) df_mentions = pd.read_csv('gdrive/My Drive/corona/Data/df_mentions_alldat.csv', index_col=[0])  import ast df_mentions['mentions'] = df_mentions['mentions'].apply(ast.literal_eval)  df_mentions  df_mentions = df_mentions.explode('mentions')  df_mentions  df_mentions = df_mentions.dropna() df_mentions  df_mentions.info() Social network graphs !pip install igraph &> /dev/null !pip install networkx &> /dev/null !pip install tqdm &> /dev/null Nodes for each dominant topics df1 = df.copy() df1 = df1.dropna()  df1 = df1[["dominant_topic", "tidy_tweet"]]  t0df1 = df1[(df1["dominant_topic"] == 0)] t1df1 = df1[(df1["dominant_topic"] == 1)] t2df1 = df1[(df1["dominant_topic"] == 2)] t3df1 = df1[(df1["dominant_topic"] == 3)] t4df1 = df1[(df1["dominant_topic"] == 4)]  t0mask = t0df1['tidy_tweet'].str.contains('trump\|need\|help\|state\|health\|hospital\|mask\|response\|people\|care')  t1mask = t1df1['tidy_tweet'].str.contains('time\|thank\|family\|love\|hope\|happy\|california\|together\|great\|friend')  t2mask = t2df1['tidy_tweet'].str.contains('home\|stay\|people\|going\|back\|like\|work\|need\|time\|week')  t3mask = t3df1['tidy_tweet'].str.contains('today\|time\|like\|good\|night\|going\|first\|hair\|made\|morning') t4mask = t4df1['tidy_tweet'].str.contains('people\|like\|would\|trump\|know\|virus\|death\|case\|think\|could')   t0df1 = t0df1[t0mask] t1df1 = t1df1[t1mask] t2df1 = t2df1[t2mask] t3df1 = t3df1[t3mask] t4df1 = t4df1[t4mask]  t4df1  import pandas as pd import itertools  t0word_stems = ["trump","need","help","state","health","hospital","mask","response","people","care"] t1word_stems = ["time","thank","family","love","hope","happy","california","together","great","friend"] t2word_stems = ["home","stay","people","going","back","like","work","need","time","week"] t3word_stems = ["today","time","like","good","night","going","first","hair","made","morning"] t4word_stems = ["people","like","would","trump", "know","virus","death","case","think","could"]  t0nodes = pd.DataFrame(t0word_stems, columns=['label']) t1nodes = pd.DataFrame(t1word_stems, columns=['label']) t2nodes = pd.DataFrame(t2word_stems, columns=['label']) t3nodes = pd.DataFrame(t3word_stems, columns=['label']) t4nodes = pd.DataFrame(t4word_stems, columns=['label'])  t0nodes.to_csv("topic 0 nodes.csv", index= False) t1nodes.to_csv("topic 1 nodes.csv", index= False) t2nodes.to_csv("topic 2 nodes.csv", index= False) t3nodes.to_csv("topic 3 nodes.csv", index= False) t4nodes.to_csv("topic 4 nodes.csv", index= False)  from google.colab import files files.download('topic 0 nodes.csv')  from google.colab import files files.download('topic 1 nodes.csv')  from google.colab import files files.download('topic 2 nodes.csv')  from google.colab import files files.download('topic 3 nodes.csv')  from google.colab import files files.download('topic 4 nodes 2.csv')  t0df1['tidy_tweet_list'] = t0df1['tidy_tweet'].apply(lambda x: x.split()) t1df1['tidy_tweet_list'] = t1df1['tidy_tweet'].apply(lambda x: x.split()) t2df1['tidy_tweet_list'] = t2df1['tidy_tweet'].apply(lambda x: x.split()) t3df1['tidy_tweet_list'] = t3df1['tidy_tweet'].apply(lambda x: x.split()) t4df1['tidy_tweet_list'] = t4df1['tidy_tweet'].apply(lambda x: x.split()) Edges for each dominant topic t4df1  #only keep words in word stems list t0df1["reduced"] = t0df1['tidy_tweet_list'].apply(lambda x: [item for item in x if item in t0word_stems]) t1df1["reduced"] = t1df1['tidy_tweet_list'].apply(lambda x: [item for item in x if item in t1word_stems]) t2df1["reduced"] = t2df1['tidy_tweet_list'].apply(lambda x: [item for item in x if item in t2word_stems]) t3df1["reduced"] = t3df1['tidy_tweet_list'].apply(lambda x: [item for item in x if item in t3word_stems]) t4df1["reduced"] = t4df1['tidy_tweet_list'].apply(lambda x: [item for item in x if item in t4word_stems])  t4df1  t0df1['reduced'] = [' '.join(map(str, l)) for l in t0df1['reduced']] count = t0df1['reduced'].str.split().str.len() #only keep lists with more than 1 words in the list (only keep words with a connection) t0df1 = t0df1[~(count<=1)] t0df1["reduced"] = t0df1["reduced"].apply(lambda x: x.split())  t1df1['reduced'] = [' '.join(map(str, l)) for l in t1df1['reduced']] count = t1df1['reduced'].str.split().str.len() #only keep lists with more than 1 words in the list (only keep words with a connection) t1df1 = t1df1[~(count<=1)] t1df1["reduced"] = t1df1["reduced"].apply(lambda x: x.split())  t2df1['reduced'] = [' '.join(map(str, l)) for l in t2df1['reduced']] count = t2df1['reduced'].str.split().str.len() #only keep lists with more than 1 words in the list (only keep words with a connection) t2df1 = t2df1[~(count<=1)] t2df1["reduced"] = t2df1["reduced"].apply(lambda x: x.split())  t3df1['reduced'] = [' '.join(map(str, l)) for l in t3df1['reduced']] count = t3df1['reduced'].str.split().str.len() #only keep lists with more than 1 words in the list (only keep words with a connection) t3df1 = t3df1[~(count<=1)] t3df1["reduced"] = t3df1["reduced"].apply(lambda x: x.split())  t4df1['reduced'] = [' '.join(map(str, l)) for l in t4df1['reduced']] count = t4df1['reduced'].str.split().str.len() #only keep lists with more than 1 words in the list (only keep words with a connection) t4df1 = t4df1[~(count<=1)] t4df1["reduced"] = t4df1["reduced"].apply(lambda x: x.split())  t4df1  #combination (nC2) def combine(row):  comb_list = []  for comb in itertools.combinations(row, 2):  comb_list.append(comb)  return comb_list  t0df1["comb"] = t0df1["reduced"].apply (lambda row: combine(row)) t1df1["comb"] = t1df1["reduced"].apply (lambda row: combine(row)) t2df1["comb"] = t2df1["reduced"].apply (lambda row: combine(row)) t3df1["comb"] = t3df1["reduced"].apply (lambda row: combine(row)) t4df1["comb"] = t4df1["reduced"].apply (lambda row: combine(row))  t4df1  from itertools import chain edges_df_topic0 = pd.DataFrame(list(chain.from_iterable(t0df1.comb)), columns=['from','to']).reset_index(drop=True) edges_df_topic0.head()  from itertools import chain edges_df_topic1 = pd.DataFrame(list(chain.from_iterable(t1df1.comb)), columns=['from','to']).reset_index(drop=True) edges_df_topic1.head()  from itertools import chain edges_df_topic2 = pd.DataFrame(list(chain.from_iterable(t2df1.comb)), columns=['from','to']).reset_index(drop=True) edges_df_topic2  from itertools import chain edges_df_topic3 = pd.DataFrame(list(chain.from_iterable(t3df1.comb)), columns=['from','to']).reset_index(drop=True) edges_df_topic3  from itertools import chain edges_df_topic4 = pd.DataFrame(list(chain.from_iterable(t4df1.comb)), columns=['from','to']).reset_index(drop=True) edges_df_topic4  edges_df_topic0.to_csv("edges word stem topic0 1.csv", index= False) from google.colab import files files.download('edges word stem topic0 1.csv')  edges_df_topic1.to_csv("edges word stem topic1 1.csv", index= False) from google.colab import files files.download('edges word stem topic1 1.csv')  edges_df_topic2.to_csv("edges word stem topic2 1.csv", index= False) from google.colab import files files.download('edges word stem topic2 1.csv')  edges_df_topic3.to_csv("edges word stem topic3 1.csv", index= False) from google.colab import files files.download('edges word stem topic3 1.csv')  edges_df_topic4.to_csv("edges word stem topic4 1.csv", index= False) from google.colab import files files.download('edges word stem topic4 2.csv') |
| social network graphs and centrality measures | network word stem topic revised.R  library(dplyr)  library(igraph)  library(tibble)  #topic 0  nodes = read.csv(file = 'topic 0 nodes.csv', na.strings=c("","NA"))  per_stem = read.csv(file = 'edges word stem topic0 1.csv', na.strings=c("","NA"))  colnames(per_stem) = c("stem1", "stem2")  per_stem <- per_stem %>%  group_by(stem1, stem2) %>%  summarise(weight = n()) %>%  ungroup()  View(per_stem)  nodes <- nodes %>% rowid_to_column("id")  View(nodes)  edges <- per_stem %>%  left_join(nodes, by = c("stem1" = "label")) %>%  rename(from = id)  edges <- edges %>%  left_join(nodes, by = c("stem2" = "label")) %>%  rename(to = id)  edges <- select(edges, from, to, weight)  network <- graph_from_data_frame(d = edges, vertices = nodes, directed = F)  network <- simplify(network, remove.multiple=F, remove.loops=T)  V(network)$size <- edges$weight*0.2  E(network)$color <- as.factor(edges$to)  # Make the plot  plot(network,  vertex.label= V(network)$label,  vertex.color = '#f7786f',  vertex.label.color = "black",  vertex.frame.color= "black",  vertex.label.dist=0,  vertex.label.cex = 2.5,  vertex.label.font = 2,  edge.width = 0.5,  edge.color = "grey",  layout = layout_nicely)  #topic 1  nodes = read.csv(file = 'topic 1 nodes.csv', na.strings=c("","NA"))  per_stem = read.csv(file = 'edges word stem topic1 1.csv', na.strings=c("","NA"))  colnames(per_stem) = c("stem1", "stem2")  per_stem <- per_stem %>%  group_by(stem1, stem2) %>%  summarise(weight = n()) %>%  ungroup()  View(per_stem)  nodes <- nodes %>% rowid_to_column("id")  View(nodes)  edges <- per_stem %>%  left_join(nodes, by = c("stem1" = "label")) %>%  rename(from = id)  edges <- edges %>%  left_join(nodes, by = c("stem2" = "label")) %>%  rename(to = id)  edges <- select(edges, from, to, weight)  network <- graph_from_data_frame(d = edges, vertices = nodes, directed = F)  network <- simplify(network, remove.multiple=F, remove.loops=T)  V(network)$size <- edges$weight*0.7  E(network)$color <- as.factor(edges$to)  # Make the plot  plot(network,  vertex.label= V(network)$label,  vertex.color = '#a2d67e',  vertex.label.color = "black",  vertex.frame.color= "black",  vertex.label.dist=0,  vertex.label.cex = 2.5,  vertex.label.font = 2,  edge.width = 0.5,  edge.color = "grey",  layout = layout_nicely)  #topic 2  nodes = read.csv(file = 'topic 2 nodes.csv', na.strings=c("","NA"))  per_stem = read.csv(file = 'edges word stem topic2 1.csv', na.strings=c("","NA"))  colnames(per_stem) = c("stem1", "stem2")  per_stem <- per_stem %>%  group_by(stem1, stem2) %>%  summarise(weight = n()) %>%  ungroup()  View(per_stem)  nodes <- nodes %>% rowid_to_column("id")  View(nodes)  edges <- per_stem %>%  left_join(nodes, by = c("stem1" = "label")) %>%  rename(from = id)  edges <- edges %>%  left_join(nodes, by = c("stem2" = "label")) %>%  rename(to = id)  edges <- select(edges, from, to, weight)  network <- graph_from_data_frame(d = edges, vertices = nodes, directed = F)  network <- simplify(network, remove.multiple=F, remove.loops=T)  V(network)$size <- edges$weight*0.12  E(network)$color <- as.factor(edges$to)  # Make the plot  plot(network,  vertex.label= V(network)$label,  vertex.color = '#7ed6d3',  vertex.label.color = "black",  vertex.frame.color= "black",  vertex.label.dist=0,  vertex.label.cex = 2.5,  vertex.label.font = 2,  edge.width = 0.5,  edge.color = "grey",  layout = layout_nicely)  #topic 3  nodes = read.csv(file = 'topic 3 nodes.csv', na.strings=c("","NA"))  per_stem = read.csv(file = 'edges word stem topic3 1.csv', na.strings=c("","NA"))  colnames(per_stem) = c("stem1", "stem2")  per_stem <- per_stem %>%  group_by(stem1, stem2) %>%  summarise(weight = n()) %>%  ungroup()  View(per_stem)  nodes <- nodes %>% rowid_to_column("id")  View(nodes)  edges <- per_stem %>%  left_join(nodes, by = c("stem1" = "label")) %>%  rename(from = id)  edges <- edges %>%  left_join(nodes, by = c("stem2" = "label")) %>%  rename(to = id)  edges <- select(edges, from, to, weight)  network <- graph_from_data_frame(d = edges, vertices = nodes, directed = F)  network <- simplify(network, remove.multiple=F, remove.loops=T)  V(network)$size <- edges$weight*0.1  E(network)$color <- as.factor(edges$to)  # Make the plot  plot(network,  vertex.label= V(network)$label,  vertex.color = '#b58bc9',  vertex.label.color = "black",  vertex.frame.color= "black",  vertex.label.dist=0,  vertex.label.cex = 2.5,  vertex.label.font = 2,  edge.width = 0.5,  edge.color = "grey",  layout = layout_nicely)  #topic 4  nodes = read.csv(file = 'topic 4 nodes 2.csv', na.strings=c("","NA"))  per_stem = read.csv(file = 'edges word stem topic4 2.csv', na.strings=c("","NA"))  colnames(per_stem) = c("stem1", "stem2")  per_stem <- per_stem %>%  group_by(stem1, stem2) %>%  summarise(weight = n()) %>%  ungroup()  #View(per_stem)  nodes <- nodes %>% rowid_to_column("id")  #View(nodes)  edges <- per_stem %>%  left_join(nodes, by = c("stem1" = "label")) %>%  rename(from = id)  edges <- edges %>%  left_join(nodes, by = c("stem2" = "label")) %>%  rename(to = id)  edges <- select(edges, from, to, weight)  network <- graph_from_data_frame(d = edges, vertices = nodes, directed = F)  network <- simplify(network, remove.multiple=F, remove.loops=T)  V(network)$size <- edges$weight*0.05  E(network)$color <- as.factor(edges$to)  # Make the plot  plot(network,  vertex.label= V(network)$label,  vertex.color = '#fcb68b',  vertex.label.color = "black",  vertex.frame.color= "black",  vertex.label.dist=0,  vertex.label.cex = 2.5,  vertex.label.font = 2,  edge.width = 0.5,  edge.color = "grey",  layout = layout_nicely)  #centrality measures  #degree centrality  degree.cent <- degree(network, mode = "all", loops=FALSE, v=V(network))  degree.cent  #betweenness  betweenness.cent <- betweenness(network, v=V(network), directed=FALSE)  betweenness.cent  #closeness  closeness.cent <- closeness(network, mode="all", vids = V(network))  closeness = data.frame(closeness.cent)  #eigenvector centrality  eigen.cent <- eigen_centrality(network, directed=FALSE)  eigen = data.frame(eigen.cent$vector)  #size  gsize(network)  #density  edge_density(network, loops = FALSE)  #diameter  diameter(network, directed = FALSE) |
| State Heatmap | State_heatmap for paper revised.R  df = read.csv(file = 'state_df3.csv', na.strings=c("","NA"))  library(viridis)  library(usmap)  library(ggplot2)  plot_usmap(data = df, values = "compound_score", color = "white", labels=TRUE, label_color="white") +  theme(legend.position = "right")+  ggtitle("") +  scale_fill_gradient(low = "grey47",  high = "red",  name = "Average Sentiment Score",  breaks = c(0.10, 0.14, 0.18)) +  theme(text=element_text(size=22, face="bold", colour="black"),  plot.title = element_text(hjust = 0.5)) |
